# Exposure to Polycyclic Aromatic Hydrocarbons Leads to Non-monotonic Modulation of DNA and RNA (hydroxy)methylation in a Rat Model

**DOI:** 10.1038/s41598-018-28911-y

**Published:** 2018-07-12

**Authors:** Radu-Corneliu Duca, Nathalie Grova, Manosij Ghosh, Jean-Mikael Do, Peter H. M. Hoet, Jeroen A. J. Vanoirbeek, Brice M. R. Appenzeller, Lode Godderis

**Affiliations:** 1 Centre for Environment and Health, Department of Public Health and Primary Care, University of Leuven (KU Leuven), Kapucijnenvoer 35 blok D, box 7001, 3000 Leuven, Belgium; 2 Human Biomonitoring Research Unit, Luxembourg Institute of Health, rue Henri Koch 29, 4354 Esch-sur-Alzette, Luxembourg; 3 External Service for Prevention and Protection at Work, IDEWE, Interleuvenlaan 58, 3001 Heverlee, Belgium

## Abstract

Besides genetic modifications, rapidly growing evidence has linked environmental pollutants with epigenetic variations. To date, only a few studies have been performed on DNA methylation changes of polycyclic aromatic hydrocarbons (PAH), which showed contradictory results. These discrepancies might be partially explained by differences in used agents. Generally in *in vitro* studies, a single compound is used, while in humans environmental studies, multi-residue exposure is investigated. The present study aimed to study epigenetic alterations induced by multi-residue exposure to PAH. Female Long Evans rats were exposed to a mixture of 16 US-EPA priority PAH, 3 times per week over a 90-day period. The livers were used to assess the (hydroxy)methylation status of genomic DNA/RNA, together with reduced and oxidized forms of glutathione. The results of this study demonstrate that a multi-residue exposure to PAH affects glutathione status, DNA (hydroxy)methylation, and RNA (hydroxy)methylation, together with DNA PAH-adducts formation. In addition, a non-monotonic response relationship was demonstrated between PAH concentration, the levels of glutathione and DNA (hydroxy)methylation levels at environmental relevant doses. This hormetic response gives a novel insight concerning the toxicity of environmental pollutants such as PAH and the biological response that may be different depending on the level of exposure.

## Introduction

Polycyclic aromatic hydrocarbons (PAH) originate from unburned fuel, lubricating oil and/or are formed during incomplete combustion of organic material^1^. These environmental pollutants represent an important cause of morbidity and mortality both in the general population and in occupationally exposed individuals^2,3^. Epidemiological studies have identified PAH as important causative agents for lung, colorectal, pancreatic and prostate cancers^4–6^, yet the actual mechanisms by which pollution causes the adverse health effects is not yet completely understood.

It is accepted that environmental carcinogens induce disease pathways through changes in the genome^7,8^, which can alter the expression of specific genes^9^. Whether these changes are a response to DNA damage and/or a consequence of epigenetic modifications needs further research. Growing evidence has linked environmental pollutants with epigenetic variations, including changes in DNA methylation^10^. These mechanisms are likely to play an important role in disease etiology. Epigenetic changes described in relation to environmental exposure were similar to changes observed in chronic diseases, such as cancer. For instance, aberrant 5-methyl cytosine levels, like global hypomethylation and/or gene-specific hypermethylation or hypomethylation, were observed in diseases like leukemia, prostate cancer, hepatic cancer^11,12^. Interestingly, similar changes have also been observed in cells exposed to carcinogenic agents^11,13,14^. A better understanding might open options for disease prevention and follow up through the development of biomarkers reflecting exposures to environmental pollutants and/or predicting the risk of future disease.

To date, research concerning the effect of PAH exposure on the global DNA methylation are rather limited and contradictory. For instance, no global DNA methylation changes were observed in an *in vitro* study using TK6 cells exposed to benzo[a]fluoranthene, benzo[a]pyrene (B[a]P) or benzo[a]anthracene^9^, while other *in vitro* studies showed that exposure to B[a]P induced global DNA hypomethylation in zebrafish embryos^15^ and global DNA hypermethylation in mouse embryonic fibroblasts^16^. Furthermore, cultured primary cell lines of human bronchial epithelial cells treated with B[a]P showed alterations in DNA methylation, including DNA hypomethylation and hypermethylation^17–19^.

Also, differences in DNA methylation levels have been reported in peripheral blood lymphocytes of workers chronically exposed to PAH compare to their matched controls^16,20^ and were further correlated with the benzo[a]pyrene diolepoxide - DNA adducts and 1-hydroxypyrine levels in urine of occupationally exposure persons^20^. In a birth cohort study, a lower degree of placental global DNA methylation in early pregnancy was associated with increased urban air exposure to particulate matter^21^. Unlike for particulate matter, the exposure to PAH in air during pregnancy has been shown to be associated with higher levels of cord blood global DNA methylation, which was further associated with the presence of DNA adducts^22^.

The differences between *in vitro*, *in vivo* and human studies can partially be explained by differences in exposure and dosing. While *in vitro* and *in vivo* studies, mostly use a single compound exposure, aiming to evaluate the detrimental effects of a single specific compound (e.g. B[a]P), it is inherit to environmental exposure in humans’ a combined exposure to multiple chemicals occurs. Furthermore, often relatively high levels of exposure and dosing are investigated in animal or *in vitro* studies, which are less representative of environmental or occupational human exposure. In this context, we investigated epigenetic modification, including (hydroxy)methylation status of genomic DNA and RNA, together with the reduced and oxidized forms of the glutathione, induced by a multi-residue exposure to PAH, in a rat model.

## Materials and Methods

### Animal Experimentation

#### Animal housing

Sixty-four Long Evans rats (female of 180–200 g, Elevage Janvier, St Berthevin, France) were housed in plastic cages under controlled environment (12 h light/dark cycle, light on at 7 am, temperature of 22 ± 2 °C and relative humidity of 40 ± 5%), in the animal facilities of Luxembourg Institute of Health, Grand-Duchy of Luxembourg. Food and water were available *ad libitum*. The water, food and oil were tested according to NF ISO 15302 to confirm that all these matrices were PAH-free down to a detection limit of 10 ng/L of water and 1 ng/g of fat. Rats were acclimatized to the animal facility for 2 weeks prior to experiment start.

#### Animal treatment

The mix of PAH was composed of the 16 compounds pointed out by the US-Environmental Protection Agency for their toxicity (Table 1). A mix was prepared in vegetable oil weekly (ISIO4, Lesieur, Neuilly-sur-Seine, France). Eight rats were randomly allocated to each of the experimental groups receiving 10, 20, 40, 80, 200, 400 or 800 µg/kg body weight of each compound included in the mix, by gavage, 3 times per week over a 90-day period. The lowest level corresponds to the lowest levels allowing metabolites of PAH detection in rats hair after 1-month exposure, based on previous study^23^. The highest level of exposure was based on the US-EPA estimated level of carcinogenic risk for B[a]P corresponding to 1/15 of the 36.5 mg /kg b.w./week oral exposure^24^. Control rats received the vehicle, vegetable oil, only. At the end of the 90 days-experiment, the rats were euthanized by using carbon dioxide. All procedures were conducted in compliance with European Communities Council Directive of 22 September 2010 (2010/63/EU) and authorized by the Ministry of Agriculture, Grand-Duchy of Luxembourg.

**Table 1:** Chemical structures of the 16 US-EPA priority PAHs used for rats treatment.

Compound	Molecular Formula	Structural Formula	Molecuer weight (g/mol)	CAS No.
Naphthalene	C_10_H_8_		128,1	91-20-3
Acenaphthylene	C_12_H_8_		152,1	208-96-8
Acenaphtene	C_12_H_10_		154,2	83-32-9
Fluorene	C_13_H_10_		166,2	86-73-7
Anthracene	C_14_H_10_		178,2	120-12-7
Phenanthrene	C_14_H_10_		178,2	85-01-8
Fluoranthene	C_16_H_10_		202,3	206-44-0
Pyrene	C_16_H_10_		202,3	129-00-0
Benz[a]anthracene	C_18_H_12_		228,3	56-55-3
Chrysene	C_18_H_12_		228,3	218-01-9
Benzo[b]fluoranthene	C_20_H_12_		252,3	205-99-2
Benzo[k]fluoranthene	C_20_H_12_		252,3	207-08-9
Benzo[a]pyrene	C_20_H_12_		252,3	50-32-8
Indeno[1,2,3-cd]pyrene	C_22_H_12_		276,3	193-39-5
Benzo[g,h,i]perylene	C_22_H_12_		276,3	191-24-2
Dibenz[a,h]anthracene	C_22_H_14_		278,4	53-70-3

#### Samples collections

Urine samples were collected for 24 h prior to day 90. Immediately after gavage, the rats were placed in individual metabolic cages (Type 304 Stainless steel, Techniplast, Zwaag, Netherlands) and urine was collected in refrigerated tubes and weighed before storage at − 20 °C.

Livers were dissected and weighed. The base of the left lateral liver lobe was divided into 5 equivalent pieces of 100 mg each, which were placed in cryogenic tubes, frozen in liquid nitrogen and stored at −80 °C before analysis.

### Samples analysis

#### Analysis of OH-PAH in urine samples

Fifty metabolites of PAH (mono-hydroxylated-PAH, OH-PAH), corresponding to the most common metabolized forms of the 16 PAH used for animal treatment, were assessed in urine samples using a previously published method^25^. Briefly, urine samples were incubated overnight at 37 °C in present of sulfatase and glucuronidase from Helix pomatia (Sigma-Aldrich, Bormen, Belgium) at pH 5.6. Upon acidification with HCl 32%, the samples were further purified using successively a liquid-liquid extraction with ethyl acetate/cyclohexane (50:50, v/v) and a solid phase extraction onto an Envi-Chrom P cartridge (Sigma-Aldrich, Bormen, Belgium). OH-PAH were eluted with acetate/cyclohexane (50:50, v/v), evaporated, resuspended in cyclohexane/methanol/water (50:40:10, v/v/v). The methanol-water layer containing OH-PAH was evaporated until fully dried. The residue was then further derivatized with 20 µL MtBSTFA for 30 min at 60 °C and then 1 µL was injected into an Agilent 7890 A gas chromatograph equipped with an HP-5MS capillary column (30 m, 0.25 mm i.d., 0.25 µm film thickness), coupled with an Agilent 7000B triple quadrupole mass spectrometer operating in electron impact ionization mode^26^.

#### Glutathione determination in liver samples

Glutathione (GSH) and glutathione disulfide (GSSG) concentrations in liver tissue were analyzed using a previously published method^27^. Briefly, frozen liver samples were homogenized on ice in cold 40 mM N-ethylmaleimide (20 mL/g tissue, to prevent rapid oxidation of GSH) and then centrifuged at 14000 g for 15 minutes at 4 °C. The supernatant was transferred to a new tube and 5% metaphosphoric acid was added (1/5th of the supernatant volume, final concentration is 1% metaphosphoric acid, for removing the proteins), mixed and again centrifuged at 14000 g during 15 minutes at 4 °C. The supernatant was stored at −80 °C until measurements. The measurements of the reduced and oxidized forms of glutathione were performed by ultra-pressure liquid chromatography (UPLC), in combination with tandem mass spectrometry (MS-MS). The LC/MS-MS analysis was conducted on a Waters® Acquity UPLC^TM^, coupled to a Waters® Micromass Quattro Premier^TM^ Mass Spectrometer using electro spray ionization (ESI). Data were further normalized to protein content measured by the Bradford method using bovine serum albumin as the standard. The ratio of total glutathione to glutathione disulfide was then calculated.

#### Epigenetic markers analysis

Genomic DNA/RNA was isolated from frozen liver tissue samples using the AllPrep DNA/RNA kit (Qiagen) following the manufacturer’s protocol. The DNA/RNA was quantified using a spectrophotometer Nanodrop 2000 (ND-2000). The ratio of wavelength at 260 nm and 280 nm was used to assess the purity of DNA. The isolated DNA/RNA (1 µg) was further enzymatically hydrolyzed to individual deoxyribonucleosides by a simple one-step hydrolysis procedure^28^. A digest mix was prepared by adding phosphodiesterase I, alkaline phosphatase and benzonase® Nuclease to Tris-HCl buffer. Extracted DNA/RNA was spiked with IS’s mixture, dried and then hydrolyzed at 37 °C for at least 8 h in presence of 10 µL digest mix. After hydrolysis, 490 µL ACN:H_2_O (90:8, v/v) was added to each sample. In each digested DNA/RNA sample, both DNA and RNA (hydroxy)methylation at the C5 position of cytosine (m^5^C and hm^5^C). was determined using a HILIC-UPLC-MS/MS method that was previously published elsewhere^29,30^.

#### DNA adducts analysis

Tetrahydroxylated polycyclic aromatic hydrocarbons (tetra-OH-PAH) resulting from the hydrolysis of their respective diol-epoxide precursors which are involved in DNA-adduct formation have been analyzed in DNA samples using a previously published method^26^. Briefly, 50 µg of each liver DNA sample were submitted to acidic hydrolysis (0.5 mL HCL 0.1 N) at 90 °C for 3 h. After hydrolysis, the samples were supplemented with 50 µL NaOH (1 N) and 0.5 mL phosphate buffer (1 M, pH 7) and the mixture was purified using solid phase extraction on a C18 cartridge (Sigma-Aldrich, Bormen, Belgium). Tetra-OH-PAH isomers were subsequently eluted with methanol, evaporated to dryness, derivatizated in 10 µL MSTFA for 120 min at 60 °C, and 2 µL of extract were injected into a GC–MS/MS.

### Statistical analysis

Linear models have been used to compare the outcome (e.g. GSH, GSSG, etc.) as a function of dose. When needed, a transformation (natural logarithm, ln; or square root, sqrt) was applied to obtain a more symmetric distribution of model residuals. First, a one-way ANOVA test was applied using a Graphpad 6 (Graphpad Software Inc, San Diego, USA). The global test for a difference between the dose levels has 7 degrees of freedom (8 dose levels). Bonferroni’s post-hoc tests were reported for multiple comparisons versus the control level. Second, a regression model with restricted cubic splines^31^ using a SAS software, version 9.4 of the SAS System for Windows (SAS Institute Inc., Cary, NC, USA) was applied to determine whether there is a nonlinear relation between the dose levels and the outcome. A spline basis with 5 knots was applied, so that the resulting global test for a relation between the dose and outcome has 4 degrees of freedom. Finally, the same regression model with splines was applied, but with log-transformed (log10) dose as predictor. In this case, the value five was used as level for the control dose (before transformation). For each outcome, a Spearman correlation was used to evaluate the relation between its predicted value and the predicted value for GSH/GSSH, both obtained from the regression model with splines and dose on log-scale. This Spearman correlation was based on eight pairs of values, one for each dose level.

### Data availability

All data generated or analyzed during this study are included in this published article.

## Results

### Influence of PAH exposure on rats’ weight gain and health

Rats were followed on daily basis for general status and were weighed before the administration of the treatment, 3 times per week over the 90-day period of the experiment. No significant differences were observed between the groups as for average weight or weight gain (Figure S1). Furthermore, at the end of the study, rats’ livers were weighted and no significant differences between the groups were observed as for the relative liver weight to total body weight (Figure S2).

### Urine Sample Analysis

**Figure 1:**
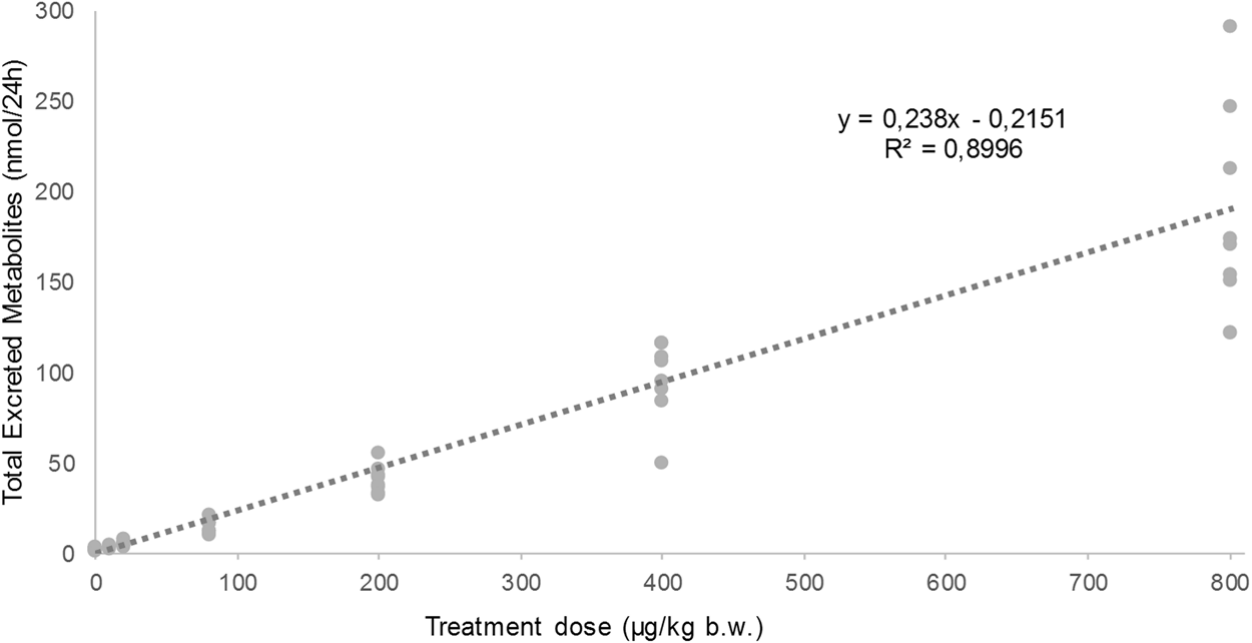
Total excreted OH-PAH in urine samples collected over 24 h from rats upon 90-days exposure to PAH mixture.

Among the 50 targeted metabolites, 41 OH-PAH were detected in urine. A detailed description of OH-PAH elimination profile has been published elsewhere^25^. Briefly, 24 of the detected metabolites corresponded to 7 parent compounds with less than 5 aromatic rings (i.e. naphthalene, fluorene, phenanthrene, pyrene, fluoranthene, B[a]A, chrysene) and 26 corresponded to 5 heavier PAH (i.e. B[a]P, B[k]F, B[b]F, I[1,2,3-c-d]P and DIB[a,h]A) out of the total of 16 PAH administered. Several OH-metabolites were also detected in the urine of the control rats suggesting background exposure of the animals by food, water or inhaled air. As showed in the Fig. 1, the sum of the detected OH-PAH in rats urine have showed a good linear correlation with the different treatment levels (R^2^ = 0.8996).

### Liver Sample Analysis

#### Glutathione modulation

**Figure 2:**
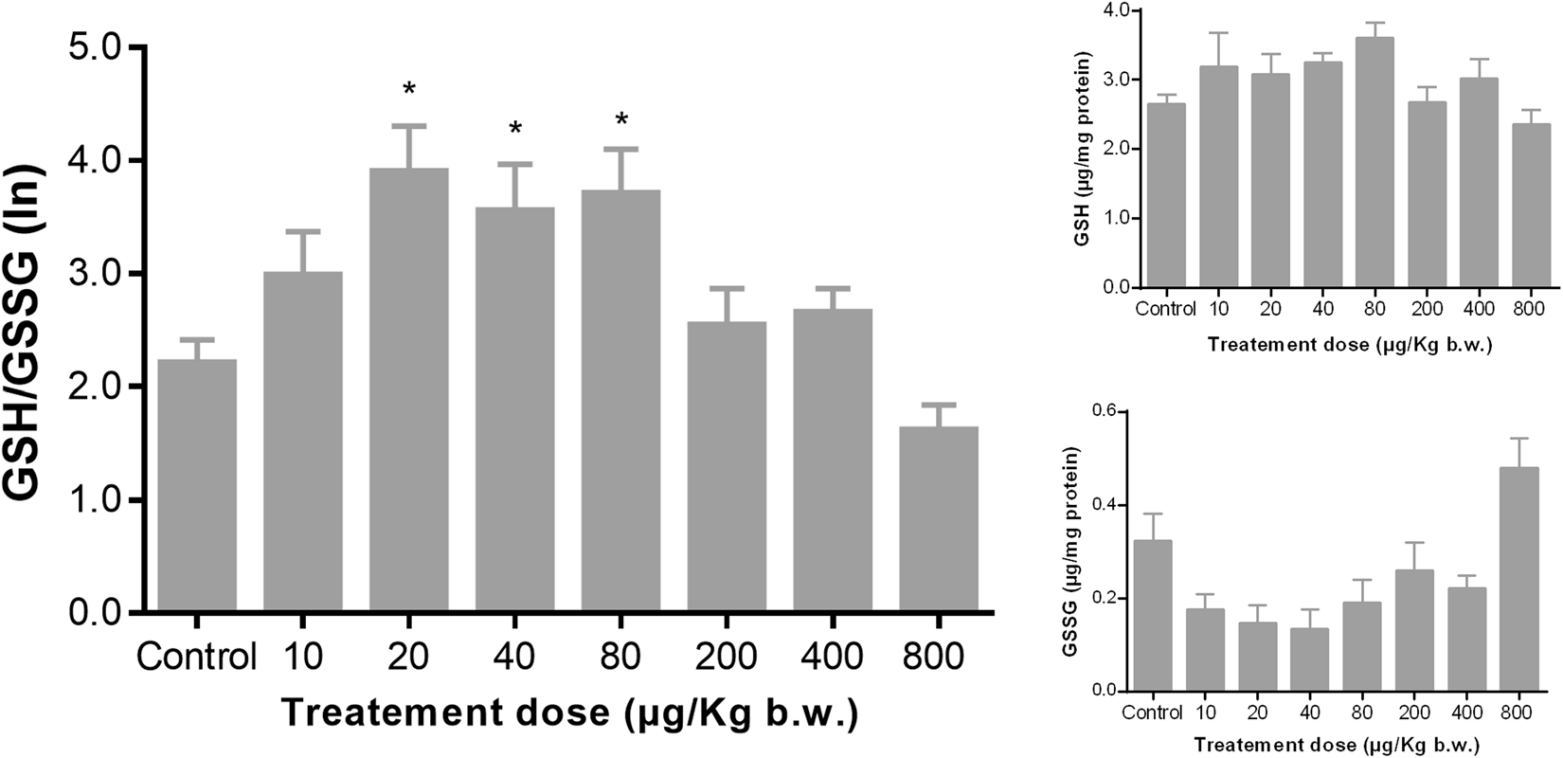
Glutathione modulation in rat liver upon exposure to PAH mixture. Glutathione and glutathione disulfide ratio (GSH/GSSG) variation (**A**), glutathione (GSH) levels variation (**B**) and disulfide glutathione (GSSG) levels variation (**C**) over different doses (n = 8, excepting for dose 40 µg/kg b.w. were n = 7) are depicted. For better visualization, to obtain a more symmetric distribution of model residuals, a natural logarithm (ln) transformation was applied for GSH/GSSG. The means together with the standard error of the mean are shown as grey columns and bars, respectively. Based on the ANOVA statistical analysis, p- values (*<0.05; ** < 0.01; ***<0.001) have been calculated using a Bonferroni’s multiple comparisons test, as detailed in the section 2.3, in order to assess the statistical significance of the observed mean values as compared to the control group.

Figure 2 shows the ratio of reduced and oxidized glutathione (GSH/GSSG) upon exposure to the PAH mixture. GSH/GSSG ratio increased in the rats treated with lower PAH concentration (i.e. 10, 20, and 40 µg/kg bw). The synthesis of reduced glutathione seems to reach a threshold at PAH treatment 40 µg/kg bw, followed by a decrease in GSH/GSSG ratio at the higher PAH treatments. Based on the ANOVA test significantly different from control were the dose levels 20, 40 and 80 µg/kg bw (p = 0.0452, 0.0154, and 0.0193, respectively). Furthermore, using the regression model, a statistically significant (F = 6.22; p = 0.0003) nonlinear relation between the treatment dose levels and the GSH/GSSG ratio was observed.

#### Global DNA (hydroxy)methylation modulation

**Figure 3:**
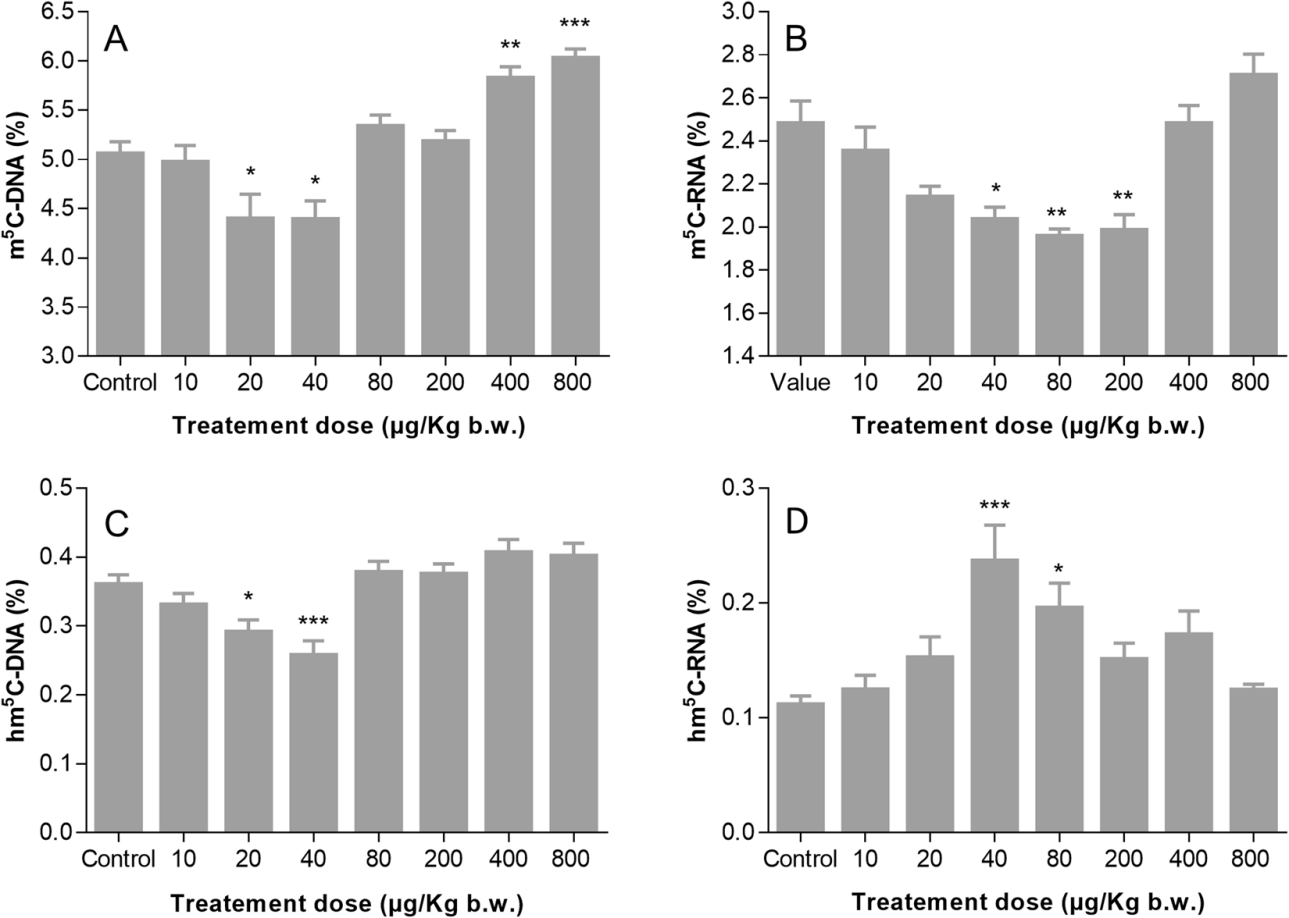
Global DNA/RNA (hydroxy)methylation modulation in rat liver upon exposure to PAH mixture. DNA methylation (m^5^C-DNA) (**A**) and hydroxymethylation (hm^5^C-DNA) (**C**) levels, RNA methylation (m^5^C-RNA) (**B**) and hydroxymethylation (hm^5^C-RNA) (**D**) levels variation over different doses (n = 8, excepting for dose 40 µg/kg b.w. were n = 7) are depicted. The means together with the standard error of the mean are shown as grey columns and bars, respectively. Based on the ANOVA statistical analysis, p- values (*<0.05; ** < 0.01; ***<0.001) have been calculated using a Bonferroni’s multiple comparisons test, as detailed in the section 2.3, in order to assess the statistical significance of the observed mean values as compared to the control group.

The average levels of m^5^C-DNA and hm^5^C-DNA varied from 4.5% to 6.0% and from 0.20% to 0.53% in DNA samples, respectively. Decreased m^5^C-DNA and hm^5^C-DNA were observed for rats exposed to lower doses (i.e. 10, 20, and 40 µg/Kg bw) (Fig. 3A,C). Based on the ANOVA test, significantly lower levels then for the control were observed at the dose levels 20 µg/kg bw (p = 0.0196, m^5^C-DNA; and p = 0.0439 hm^5^C-DNA) and 40 µg/kg bw (p = 0.0241, m^5^C-DNA; and p = 0,0004 hm^5^C-DNA). In contrast, increased levels of m^5^C-DNA and hm^5^C-DNA were observed at highest treatment levels (i.e. 400 and 800 µg/Kg bw). Based on the ANOVA test, significantly higher levels then for the control were observed only for m^5^C-DNA at the dose levels 400 µg/kg bw (p = 0.0032) and 800 µg/kg bw (p < 0.0001). In the regression model, the nonlinear relation between the treatment dose levels and the outcome were statistically significant for m^5^C-DNA (F = 8.87, p < 0.0001) but no statistical significance was found for hm^5^C-DNA (F = 0.79, p = 0.5360). Nevertheless, the levels of m^5^C-DNA and hm^5^C-DNA showed a rather good linear correlation (R^2^ = 0.61), which is in concordance with previously published findings^28^.

#### Global RNA (hydroxy)methylation modulation

The average levels of m^5^C-RNA and hm^5^C-RNA varied from 1.9% to 2.7% and 0.12% to 0.24% in RNA liver samples from rats treated with the PAH mixture, respectively. The overall relative levels of both m^5^C and hm^5^C in RNA are lower than those in DNA, which is in concordance with previously published findings^32^. The m^5^C-RNA decreased in the first three treatment groups (i.e. 10, 20, and 40 µg/Kg bw) until reaching a plateau between treatments 40 and 200 µg/kg bw (Fig. 3B,D). The m^5^C-RNA at 400 and 800 µg/kg bw was slightly higher than for control. Based on the ANOVA test, significantly lower levels of m^5^C-RNA then for the control were observed at the dose levels 40, 80 and 200 µg/kg bw (p = 0.0124, p = 0.0011 and p = 0.0017, respectively). Unlike m^5^C-RNA, hm^5^C-RNA increases in the first three treatment groups, and decreases to a lower value than the control group in the highest level. Based on the ANOVA test, significantly higher levels of hm^5^C-RNA then for the control were observed at the dose levels 40 and 80 µg/kg bw (p = 0.0002 and p = 0.0199, respectively). In a regression model, the nonlinear relation between the treatment dose levels and the outcome were statistically significant for m^5^C-RNA (F = 9.50, p < 0.0001) as well as for the hm^5^C-RNA (F = 2.69, p = 0.0426).

#### DNA adducts formation

**Figure 4:**
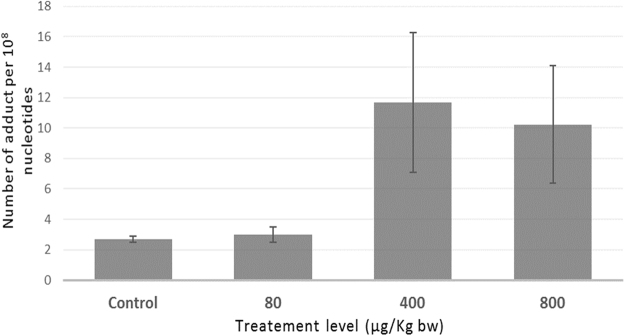
Total amount of tetrahydroxylated-PAH in liver DNA samples of rats upon exposure to the PAH mixture.

Several tetra-OH-PAH have been identified and quantified in liver DNA samples, as follows: benz[a]anthracene-r-8,t-9,c-10,t-11-tetrahydrotetrol (B[a]A-RTCT), benzo[a]pyrene-r-7,t-8,t-9,c-10-tetrahydrotetrol (B[a]P-RTTC), benzo[a]pyrene-r-7,t-8,c-9,c-10 tetrahydrotetrol (B[a]P-RTCC), phenanthrene-tetrahydrotetrol (tetra-OH-Phe) and chrysene-tetrahydrotetrol (tetra-OH-Chry-I). Figure 4 shows that the total level of tetra-OH-PAH in DNA from control rats and treated with 80 µg/Kg bw were rather similar (i.e. 2.68 ± 0.19 and 3.00 ± 0.50 adducts per 10^8^ nucleotides, respectively), whereas in DNA from 400 and 800 µg/kg were about 3–4 times higher (i.e. 11.68 ± 4.62 and 10.24 ± 3.86 adducts per 10^8^ nucleotides, respectively).

## Discussion

This study aimed to study the epigenetic alterations induced by multi-residue exposure to PAH, in order to address the previously reported contradictory results concerning DNA methylation changes of PAH. In this sense, rats have been treated systemically with a mixture of PAH at environmental relevant levels for a period of 90 days. The urinary OH-PAH concentration, revealing the elimination profile^25^, show the good linear correlation between the levels of exposure and the total amount of OH-PAH elimination, which confirms the effectiveness of the dosing. Furthermore, rat liver samples have been analyzed for reduced and oxidized glutathione and epigenetic alterations, together with DNA adducts formation. The obtained results have shown that depending on the levels of exposure, PAH toxic mechanisms changes from genomic DNA/RNA hypomethylation at lower levels to the formation of DNA adducts together with genomic DNA/RNA hypermethylation at higher levels.

Liver was chosen because of its essential role in PAH metabolism. During phase I metabolism, PAH molecules are oxidized into several epoxide metabolites that are biologically more active forms than the parent compounds^33^. These metabolites are further conjugated to glutathione during phase II metabolism, or may induce direct lesions to DNA and/or RNA. Glutathione is an important antioxidant, which can prevents cellular damage, by donating an electron to reactive oxygen species via glutathione peroxidase. Reduced glutathione (GSH) is then oxidized into glutathione disulfide molecule (GSSG). Thus, the ratio of GSH to GSSG is used as biomarker of oxidized intracellular environment^34^. Reduced ratio of GSH to GSSG is indicative of oxidative stress. Moreover, gluathathione also provides a crucial help for chemical detoxification. For instance, B[a]P has been shown to increase GSH/GSSG ratio in human hepatoma cells (HepG2). In this model, exposure to sub-lethal concentration of B[a]P caused increase in GSH and decrease in glutathione reductase activity^35^. This response acts as a defense mechanism against oxidative stress and stimulates PAH metabolites detoxification. These results are in accordance with those obtained in this study since an increase of GSH/GSSG ratio has been shown at lower levels of exposure (i.e. 10, 20, and 40 µg/Kg bw). Nevertheless, at the highest doses (i.e. 80, 200, 400 and 800 µg/Kg bw) a decrease of GSH/GSSG ratio have been observed. This non-linear modulation was mainly related to changes of GSSG levels that were statistically significant and not of GSH level more subtle modulation that were not statistically sustained. Spearman’s ranking have showed a high inverse correlation between GSH/GSSG ratio and GSH levels, which might be explained by metabolic efforts made towards the formation of GSH as detoxifying agent.

Genomic DNA hypomethylation and gene-specific hyper- and hypomethylation, were observed in diseases like cancer and have been reported in cells exposed to carcinogenic agents^28,36^. Apart from the genomic hypomethylation, several studies have found significantly lower hm5C-DNA levels in cancer cell or primary cancer tissue compared to the normal surrounding tissue^36^. In our study, decreased level of both m^5^C-DNA and hm^5^C-DNA were observed for the rats exposed to the lower doses of PAH mixture (i.e. 10, 20, and 40 µg/Kg bw). At the highest levels of treatment (i.e. 400 and 800 µg/Kg bw) only m^5^C-DNA have shown a significant increase.

Recently, it was suggested that methylated and hydroxymethylated RNA might interfere with the translational control, which provides quick adaptive responses to environmental stressors. For instance, it was shown that m^5^C-RNA could have a protective effect against tRNA degradation, which might further sustain an efficient translation^37^. Even more, hm^5^C-RNA was also shown to favour mRNA translation and to be involved in brain development in a Drosophila model^38^. Nevertheless, like for DNA hydroxymethylation, it is still not clear whether hm^5^C-RNA is a stable oxidation product or occurs transiently as an intermediate step in the pathway leading toward RNA demethylation^32^. In our study, the levels of hm^5^C-RNA increased in the first three treatment groups, following by a decrease to a lower value than the control group in the highest level. The modulation of hm^5^C-RNA is different from m^5^C-RNA as it seems that the production of hm^5^C-RNA causes a reduction of m^5^C-RNA level.

Global methylation and total tetra-OH-PAH levels in DNA samples from rats treated with the two highest levels of PAH mixture (i.e. 400 and 800 µg/Kg bw) were significantly increased as compared to control. Previously, several hypothesizes tried to explain this correlation. On one hand, PAH diol epoxides (e.g. B[a]P diol epoxide) might preferentially targeting methylated CpG sites, thus increased methylation could result in enhance PAH diol epoxide adducts formation^22^. On the other hand, the adduct formation might stimulate DNA methylation by influencing DNA methyltransferase activity in specific conditions^22,39,40^.

Previously, it has been hypothesized that an increased need of GSH, upon environmental exposure to persistent pollutants, shunt homocysteine into GSH synthesis and further reduce methionine and S-adenosylmethionine (SAM), which are a key methyl donor for the methylation of DNA, RNA and other proteins^41^. Thus, the use of a large amount of methionine for the synthesis of glutathione can cause depletion in DNA methylation and even in RNA methylation. The present results confirm this hypothesis. An increase of GSH has occurred as adaptive mechanism to the sub-chronical exposure to relatively low amounts of the 16 US-EPA PAH, potentially as response to unbalance redox environment, but especially as a phase II detoxification mechanism thought GSH conjugation. The increased need of GSH had led to depletion of DNA and RNA methylation (Rho = −0.71, p = 0.0465; and Rho = −0.88, p = 0.0039; respectively). Also, our results confirm that an increase of global DNA methylation levels at the two highest levels of exposure are positively associated with PAH DNA-adducts as previously reported^20,22^.

Thus, we further hypothesize that, depending on the levels of exposure, PAH toxic mechanisms might change from depletion of global DNA/RNA methylation at lower levels to the formation of DNA adducts at higher levels. This hypothesis is further sustained by recently published epidemiological studies intended to correlate the PAH exposure and global DNA methylation^42,43^. The differences between these recently obtained results concerning the global DNA methylation and previously published cohort studies^22,44,45^ have been explained by regional differences of human PAH exposure as reflected by urinary OH-PAH or PAH DNA-adducts^42,43^.

## Conclusion

In conclusion, these results demonstrated that a multi-residue exposure to PAH affects glutathione status, DNA and RNA (hydroxy)methylation. In addition, we found a non-monotonic response relationship between PAH concentration, glutathione levels and several epigenetic marks at environmental relevant doses, followed by the formation of PAH-adducts at higher exposure levels. This hormetic response may give insights into the knowledge concerning the toxicity of environmental pollutants such as PAH and the biological response that may be different depending on the level of exposure.

## Electronic supplementary material

Supplemental materials
