# Supplementary material for: Exposure to Polycyclic Aromatic Hydrocarbons Leads to Non-monotonic Modulation of DNA and RNA (hydroxy)methylation in a Rat Model
Source: Sci Rep. 2018 Jul 12;8:10577. doi: 10.1038/s41598-018-28911-y (PMC6043565; doi:10.1038/s41598-018-28911-y)
Supplement: Supplementary file 1 — Supplemental materials [file 41598_2018_28911_MOESM1_ESM.docx]

**Exposure to Polycyclic Aromatic Hydrocarbons Leads to Non-monotonic Modulation of DNA and RNA (hydroxy)methylation in a Rat Model**

Radu-Corneliu Duca ^1*^, Nathalie Grova ^2*^, Manosij Ghosh ^1^, Jean-Mikael Do ^1^, Peter HM Hoet ^1^, Jeroen AJ Vanoirbeek ^1^, Brice MR Appenzeller ^2**^, and Lode Godderis ^1,3**^

*co-first authors

**co-last authors

^1^ Centre for Environment and Health, Department of Public Health and Primary Care, University of Leuven (KU Leuven), Kapucijnenvoer 35 blok D, box 7001, 3000 Leuven, Belgium

^2^ Human Biomonitoring Research Unit, Luxembourg Institute of Health, rue Henri Koch 29, 4354 Esch-sur-Alzette, Luxembourg

^3^ External Service for Prevention and Protection at Work, IDEWE, Interleuvenlaan 58, 3001 Heverlee, Belgium

*Supplemental materials*

**Figure S1 – Rats average weight gain over the 90-day period of the experiment**

**Figure S2 – Rats liver relative weight after 90-day period of treatment**
